# Monocyte Activation in People With HIV and Tuberculosis Coinfection and Effect of Tuberculosis Preventive Therapy: An Analysis of the ACTG A5279/BRIEF TB Trial

**DOI:** 10.1093/ofid/ofaf771

**Published:** 2025-12-15

**Authors:** Moises A Huaman, Manuel G Feria, Michelle A Kendall, Ashley McKhann, Khuanchai Supparatpinyo, Claire A Chougnet, Xinyu Du, Frederick K Sawe, Kristine M Erlandson, Netanya S Utay, Michael M Lederman, Susan Swindells, Amita Gupta, Richard E Chaisson, Carl J Fichtenbaum

**Affiliations:** Division of Infectious Diseases, Department of Internal Medicine, University of Cincinnati College of Medicine, Cincinnati, Ohio, USA; Division of Infectious Diseases, Department of Internal Medicine, University of Cincinnati College of Medicine, Cincinnati, Ohio, USA; Center for Biostatistics in AIDS Research, Harvard T.H. Chan School of Public Health, Boston, Massachusetts, USA; Center for Biostatistics in AIDS Research, Harvard T.H. Chan School of Public Health, Boston, Massachusetts, USA; Chiang Mai University, Chiang Mai, Thailand; Division of Immunobiology, Cincinnati Children's Hospital Medical Center, Cincinnati, Ohio, USA; Center for Biostatistics in AIDS Research, Harvard T.H. Chan School of Public Health, Boston, Massachusetts, USA; Kenya Medical Research Institute/Walter Reed Project Clinical Research Center, Kericho, Kenya; Division of Infectious Diseases, Department of Medicine, University of Colorado School of Medicine, Aurora, Colorado, USA; Division of Infectious Diseases and Geographic Medicine, Department of Medicine, University of UT Southwestern Medical Center, Dallas, Texas, USA; Departments of Medicine and Pathology, Case Western Reserve University School of Medicine, Cleveland, Ohio, USA; Division of Infectious Diseases, Department of Medicine, University of Nebraska Medical Center, Omaha, Nebraska, USA; Department of Medicine, Johns Hopkins University School of Medicine, Baltimore, Maryland, USA; Department of Medicine, Johns Hopkins University School of Medicine, Baltimore, Maryland, USA; Division of Infectious Diseases, Department of Internal Medicine, University of Cincinnati College of Medicine, Cincinnati, Ohio, USA

**Keywords:** HIV, tuberculosis infection, Interferon-Gamma Release Assay, monocytes, inflammation, tuberculosis preventive therapy

## Abstract

**Background:**

Monocyte activation contributes to the pathogenesis of inflammation-driven comorbidities in people with HIV (PWH). We investigated the impact of tuberculin skin test (TST)/interferon-γ release assay (IGRA) status and tuberculosis preventive therapy (TPT) on monocyte activation in PWH.

**Methods:**

We analyzed peripheral blood mononuclear cells from participants from the A5279/BRIEF-TB trial, which compared 1 month of rifapentine/isoniazid (1HP) versus 9 months of isoniazid (9H) as TPT in PWH. All included participants were on suppressive antiretroviral therapy and had available TST or IGRA results at study entry. Samples collected at week 0 (pre-TPT) and week 48 (post-TPT) were analyzed. Monocyte subset and activation markers were measured using multiparameter flow cytometry. Proinflammatory cytokines (IL-6 and TNF-α) were assessed after 6-hour lipopolysaccharide (LPS) stimulation. Linear regression models were used for primary comparisons of monocyte markers by TST/IGRA status, adjusted for age, sex, country, and CD4 count.

**Results:**

In adjusted models, compared with TST/IGRA-negative participants (*n* = 27), TST/IGRA-positive participants (*n* = 30) had ∼2-fold relative increases in the median fluorescence intensity of CD64 (unstimulated) and CCR2 (post-LPS) on total monocytes and across monocyte subsets, pre- and post-TPT. Among TST/IGRA-positive participants, 1HP was associated with decreased fold changes over time for the percentage of CCR2+ monocytes and blunted IL-6/TNF-α responses compared with 9H.

**Conclusions:**

PWH with a positive TST or IGRA exhibited signals of monocyte activation pre- and post-TPT. TPT with 1HP led to blunted proinflammatory monocyte changes compared with 9H.

Tuberculosis remains a top infectious cause of death globally [1]. It is estimated that 1.8 billion people worldwide have been infected with *Mycobacterium tuberculosis* (*Mtb*), resulting in millions of individuals carrying a chronic, asymptomatic tuberculosis infection that could progress to tuberculosis disease [2, 3]. Although tuberculosis infection (previously known as latent tuberculosis infection) was traditionally considered a state of mycobacterial dormancy and metabolic quiescence, studies show that there may be dynamic immune-metabolic interactions between *Mtb* and the human host across the spectrum of tuberculosis infection [4–6].

Recent studies in people without HIV have shown that individuals with a positive interferon-γ release assay (IGRA) have circulating monocytes exhibiting a proinflammatory profile, which may contribute to the pathogenesis of cardiovascular diseases and other inflammation-driven comorbidities [7, 8]. These proinflammatory alterations include increased expression of monocyte receptors involved in tissue migration and cellular uptake of oxidized lipids (eg, CD36). Furthermore, we recently reported that having a positive IGRA was associated with augmented production of proinflammatory cytokines (eg, interleukin-6 [IL-6] and tumor necrosis factor-α [TNF-α]), particularly by CD14^dim^CD16^high^ nonclassical monocytes [7].

HIV infection induces a state of persistent monocyte activation. Although antiretroviral therapy (ART) overall reduces systemic inflammation, perturbations in monocyte populations and activation indices are still present in people with HIV (PWH) taking ART [9–11]. Among the underlying mechanisms of persistent monocyte activation in PWH, the presence of coinfections has emerged as a highly likely candidate [12]. However, whether coinfections, such as tuberculosis coinfection, contribute to excess monocyte activation in PWH has not been investigated thoroughly. Furthermore, the potential effects of tuberculosis preventive therapy (TPT) on monocyte profiling are unknown.

In this project, we investigated the activation profile of monocytes from PWH who participated in the Advancing Clinical Therapeutics Globally (ACTG) A5279/BRIEF TB trial (NCT01404312), a phase III trial of 4 weeks of daily rifapentine/isoniazid (1HP) versus 9 months of daily isoniazid (9H) for TPT [13]. Our primary hypothesis was that PWH with a positive tuberculin skin test (TST) or IGRA (TST/IGRA-positive) had higher levels of monocyte activation compared with TST/IGRA-negative PWH. We also explored the effects of 1HP compared with 9H TPT on monocyte activation markers in TST/IGRA-positive PWH.

## METHODS

### A5279/BRIEF TB Study Set Up and Participant Samples for NWCS 476

We analyzed cryopreserved peripheral blood mononuclear cells (PBMCs) from PWH who participated in the A5279 trial. PBMCs were obtained pre-TPT (week 0) and post-TPT (week 48). Inclusion criteria for the A5279 trial were previously described [13] and briefly included: (1) documented HIV-1 infection; (2) lived in a tuberculosis-prevalent area (defined by an estimated or reported tuberculosis prevalence of 60/100 000 inhabitants) OR had a positive test for latent tuberculosis infection; (3) absolute neutrophil count >750 cells/mm^3^, hemoglobin ≥7.4 g/dL, platelet count ≥50 000/mm^3^, liver aminotransferases ≤3 X upper limit of normal (ULN), total bilirubin ≤2.5 X ULN; (4) chest imaging without evidence of active tuberculosis; (5) not pregnant or breastfeeding with no intent to become pregnant; (6) age ≥13 years; (7) weight ≥30 kg. Additional inclusion criteria for this NWCS 476 project were: (1) on ART with HIV RNA ≤200 copies/mL at study entry; (2) result of TST or IGRA available at study entry; (3) available PBMC samples from week 0 or week 48.

Exclusion criteria for this NWCS 476 project were the same exclusionary criteria as for the parent A5279 study. Briefly, (1) treatment of tuberculosis infection within 2 years prior to study entry or presence of confirmed or probable active tuberculosis at time of study screening; (2) history of or known exposure to multidrug resistant or extensively drug resistant tuberculosis at any time prior to study entry; (3) treatment with >14 consecutive days of rifamycin or >30 consecutive days of isoniazid within 2 years prior to study entry; (4) exclusionary ART regimen at study entry (only nucleoside/nucleotide reverse transcriptase inhibitors in combination with efavirenz or nevirapine were permitted while on rifapentine). For this NWCS 476 project, we also excluded participants who developed active tuberculosis during the trial follow-up period.

### Flow Cytometry Analyses

PBMCs were thawed and rested in RPMI 1640 (Roswell Park Memorial Institute Medium) containing 10% heat-inactivated FBS and 10 mM EDTA. About 1 × 10^6^ PBMCs were incubated for 20 minutes at room temperature with Zombie UV (Biolegend), followed by Fc receptor blockage. Cells were then incubated for 30 minutes with optimized doses and combinations of mouse antihuman antibodies (Supplementary Table 1), including markers of monocyte activation (HLA-DR, CD64, CD80, and CD86), chemotaxis (CX3CR1 and CCR2), and scavenger receptors (CD36 and CD163). For in vitro stimulation assays, PBMCs were stimulated with 1 μg/mL of *Escherichia coli* lipopolysaccharide 055:B5 (LPS; Invivogen) and incubated for 6 hours at 37°C in 5% CO_2_, in the presence of 10 μg/mL of Brefeldin A. Cells were then washed with 2 mL of 1× PBS (Biolegend). Standardized doses of anti-IL-6 Alexafluor 700 (Thermo Fisher) and anti-TNF-α Brilliant Violet 650 (Mab11, Biolegend) were added and incubated for 30 minutes at 4°C.

Following antibody staining, cells were acquired on an LSR Fortessa cytometer (BD) using FACS Diva, version 6.0. At least 50 000 CD14^+^ events were acquired per sample. Flow cytometry data were then analyzed using FlowJo software, version 10.8.1 (Tree Star, Inc., Ashland, OR, USA). Fluorescence minus one controls were included to define positive thresholds. Samples were analyzed alongside aliquots of cryopreserved PBMCs from the same blood bank donor collected at the Hoxworth Blood Center in Cincinnati, Ohio, to check for batch effect. Flow cytometry data were normalized using the R package CytoNorm and FlowJo.

We used size, granularity, and fluorophore-conjugated antibody panels to distinguish monocyte populations, following a previously validated gating strategy [7]. The percentage of human monocyte subsets was determined by CD14 and CD16 expression (CD14^high^CD16^low^ classical, CD14^dim^CD16^high^ nonclassical, and CD14^high^CD16^dim^ intermediate monocytes; Supplementary Figure 1). All flow cytometry and gating analyses were carried out blinded from TST/IGRA status and TPT treatment groups.

### Objectives

The primary objective of this analysis was to compare the effect of having a positive TST or IGRA on immune activation in PWH. The exploratory objective was to examine the effect of TPT on immune activation in PWH with a positive TST or IGRA.

### Statistical Methods

The analysis population consisted of A5279/NWCS 476-eligible participants with data at either week 0 (pre-TPT) or week 48 (post-TPT). Primary comparisons were between TST/IGRA-positive (evidence of tuberculosis infection) and TST/IGRA-negative groups. If a participant received both a TST and IGRA test and only one of the tests was positive, the participant was assigned to the TST/IGRA-positive group. Additional comparisons were between the A5279 randomized treatment arms: 9H (defined as eligible participants randomized to 9 months of daily isoniazid) and 1HP (defined as eligible participants randomized to 1 month of daily isoniazid + rifapentine).

Change over time was calculated as the fold change of week 48/week 0. The relationship between TST/IGRA status and monocyte markers was assessed using multivariable linear regression models of log_10_-transformed monocyte markers (at week 0, week 48, and change over time) adjusted for age, sex at birth, country, and continuous CD4+ T cell count at study entry. The models produced fold change comparisons between groups at week 0 and week 48, and relative fold change comparisons between groups for change over time. Additionally, change over time was assessed between treatment groups and monocyte markers among those in the TST/IGRA-positive group. Figures summarizing fold changes contained the adjusted regression coefficients with their 95% confidence intervals and *P* values. *P* values <.05 were considered statistically significant. All *P* values were two-tailed. There were no adjustments for multiple testing in this exploratory analysis. All statistical analyses were performed using SAS version 9.4 and SAS/STAT version 15.2 (SAS Institute, Inc, Cary, North Carolina, USA).

### Ethical Considerations

The A5279 protocol had been approved by the institutional review board (IRB) at each site. All participants had provided written, informed consent before being involved in any research procedures. The NWCS 476 protocol was reviewed and approved by the University of Cincinnati IRB as well as by each site that provided participant samples for this analysis.

## RESULTS

PBMC samples and data of 57 participants from 4 countries were included: Brazil (4%), Kenya (18%), Thailand (42%), and USA (37%). Median age was 38 years (1st interquartile, 3rd interquartile: 34, 47). There were 33 (58%) men and 24 (42%) women. Thirty participants were categorized as TST/IGRA-positive and 27 as TST/IGRA-negative at baseline. See Table 1 for demographic and clinical characteristics of the participants. Because of the observed differences in baseline characteristics by TST/IGRA status, we present adjusted results of comparisons using regression models that controlled for age, sex at birth, country, and CD4+ T cell counts at entry.

**Table 1.** Baseline Demographic and Clinical Characteristics [Median (First Quartile and Third Quartile) or *N* (%)]

	TST/IGRA Positive (*N* = 30)	TST/IGRA Negative (*N* = 27)	Total (*N* = 57)	*P* Value^a^
Age (y)	38 (35, 48)	37 (33, 46)	38 (34, 47)	.54
Female sex at birth^b^	7 (23%)	17 (63%)	24 (42%)	.002
Race/ethnicity				<.001
White non-Hispanic	1 (3%)	0 (0%)	1 (2%)	
Black non-Hispanic	9 (30%)	9 (33%)	18 (32%)	
Hispanic (regardless of race)	13 (43%)	0 (0%)	13 (23%)	
Asian, Pacific Islander	7 (23%)	18 (67%)	25 (44%)	
Country of enrollment				<.001
Brazil	2 (7%)	0 (0%)	2 (4%)	
Kenya	1 (3%)	9 (33%)	10 (18%)	
Thailand	6 (20%)	18 (67%)	24 (42%)	
USA	21 (70%)	0 (0%)	21 (37%)	
History of comorbidities^c^	11 (37%)	8 (30%)	19 (33%)	.57
CD4+ count (cells/mm³)	639 (468, 816)	395 (319, 461)	465 (372, 650)	<.001
A5279 randomized treatment				.36
1HP	13 (46%)	15 (54%)	29 (51%)	
9H	17 (59%)	12 (41%)	28 (49%)	

^a^Continuous measures compared using a Wilcoxon Sign Rank test; categorical measures compared using a χ^2^ test.

^b^Sex at birth; gender identity not included in A5279.

^c^Comorbidities included: hypertension, diabetes, cardiovascular disease, dyslipidemia, opportunistic infections, chronic hepatitis B, chronic hepatitis C, cancer, HIV-associated mild neurocognitive disorder/HIV dementia, and chronic kidney disease/HIV-associated nephropathy.

### TST/IGRA-positive individuals had increased expression of the monocyte activation marker CD64 pre- and post-TPT, when compared with CD64 expression among TST/IGRA-negative individuals

In adjusted analyses, there were no significant differences in the percentage of total monocytes from PBMCs nor the percentages of monocyte subsets by TST/IGRA status, pre- and post-TPT (Table 2).

**Table 2.** Percentage of Total Monocytes and Monocyte Subsets by TST/IGRA Status

Study Time Point	Monocyte Subset	Percentage Of Monocyte Subset		Adjusted *P* Value^a^
		TST/IGRA Negative (*n* = 30)	TST/IGRA Positive (*n* = 27)	
Week 0	Classical	81.9%	76.9%	.21
	Intermediate	6.9%	9.5%	.33
	Nonclassical	9.3%	10.8%	.34
Week 48	Classical	80.4%	74.3%	.62
	Intermediate	8.0%	9.9%	.46
	Nonclassical	10.3%	13.2%	.32

^a^Results of comparison by TST/IGRA status after adjusting for age, sex at birth, country, and CD4 count at study entry.

**Figure 1.**
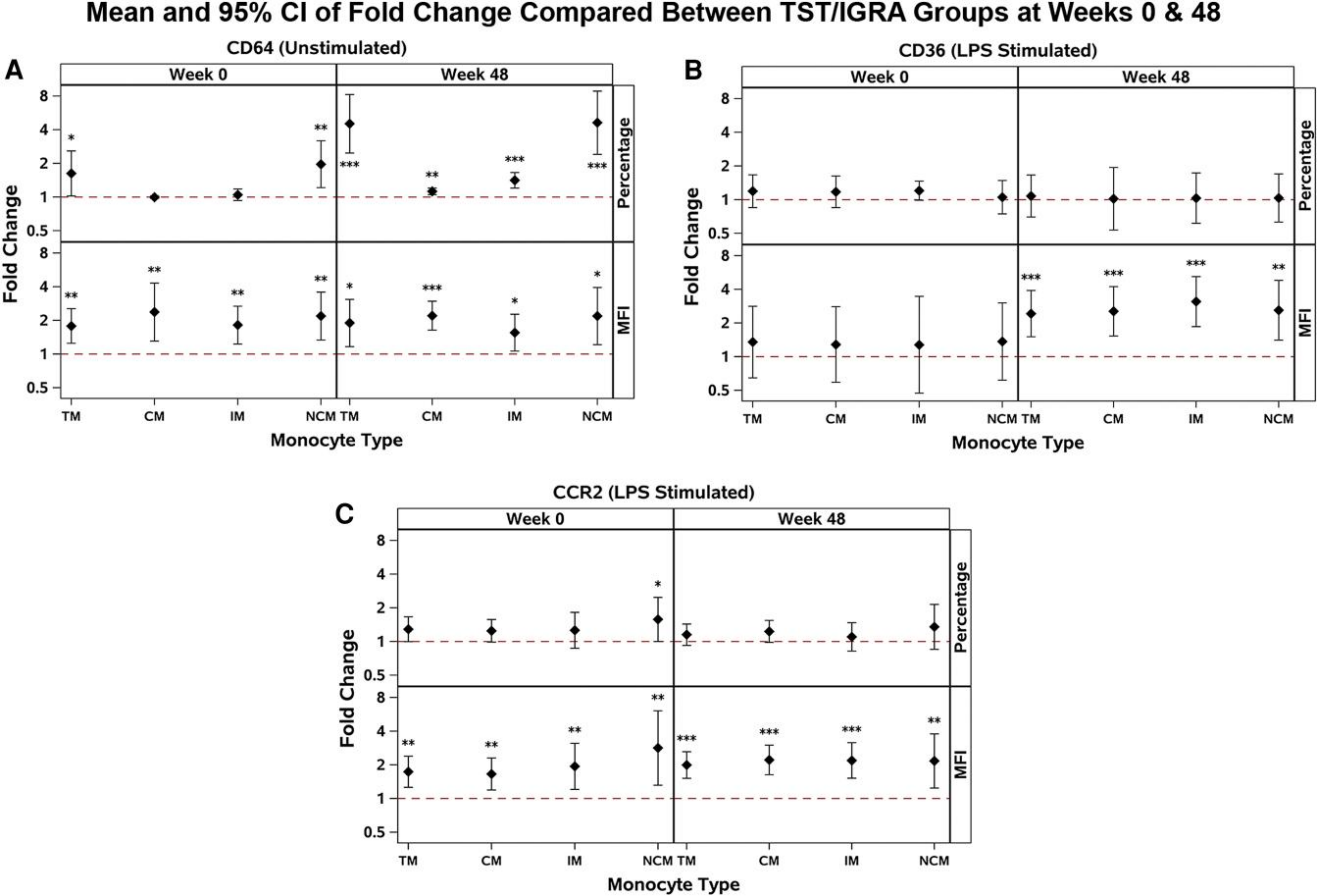
Analyses of CD64, CD36, and CCR2 expression on total monocytes and monocyte subsets between TST/IGRA-positive and TST/IGRA-negative groups at weeks 0 and 48 using multivariable linear regression models adjusted for age, sex at birth, country, and CD4+ count. The reference group is TST/IGRA-negative. Sample sizes: at week 0, *n* = 56 for CD64, CD36, and CCR2; at week 48, *n* = 53 for CD64 and *n* = 51 for CD36 and CCR2. Fold changes comparing groups for: (*A*) percentage and MFI of CD64 expression; unstimulated; (*B*) percentage and MFI of CD36 expression; after LPS stimulation; (*C*) percentage and MFI of CCR2 expression; after LPS stimulation. Abbreviations: MFI, median fluorescence intensity; LPS, lipopolysaccharide; TM, total monocytes; CM, classical monocytes; IM, intermediate monocytes; NCM, nonclassical monocytes.

Overall, we observed consistently increased expression of CD64 in TST/IGRA-positive individuals pre- and post-TPT, compared with CD64 expression among TST/IGRA-negative individuals (Supplementary Tables 2 and 3). In adjusted models, being TST/IGRA-positive was consistently associated with about a 2-fold increase in density (median fluorescence intensity, MFI) of CD64 pre- and post-TPT (Figure 1*A*).

### After LPS Stimulation, CCR2 and CD36 Expression was Higher in TST/IGRA-positive Individuals Pre- and/or Post-TPT, Than Among TST/IGRA-negative Individuals

After LPS stimulation, we observed that TST/IGRA-positive individuals (*n* = 30) had significantly higher expression of CD36 and CCR2 receptors on their monocytes, than did TST/IGRA-negative individuals (*n* = 26) (Supplementary Tables 4 and 5). In adjusted models, being TST/IGRA-positive was associated with about a 2-fold increase in density (MFI) of CD36 on total monocytes and across all monocyte subsets post-TPT (Figure 1*B*). Similarly, in adjusted models, being TST/IGRA-positive was consistently associated with about a 2-fold increase in density (MFI) of CCR2 on total monocytes and across all monocyte subsets, pre- and post-TPT (Figure 1*C*).

We did not observe statistically significant differences by TST/IGRA status in monocyte IL-6 or TNF-α production upon in vitro stimulation with LPS, at either week 0 (pre-TPT) or week 48 (post-TPT).

### TST/IGRA-positive individuals exhibited similar or more proinflammatory changes on monocyte activation markers with TPT, compared with those seen among TST/IGRA negative individuals

**Figure 2.**
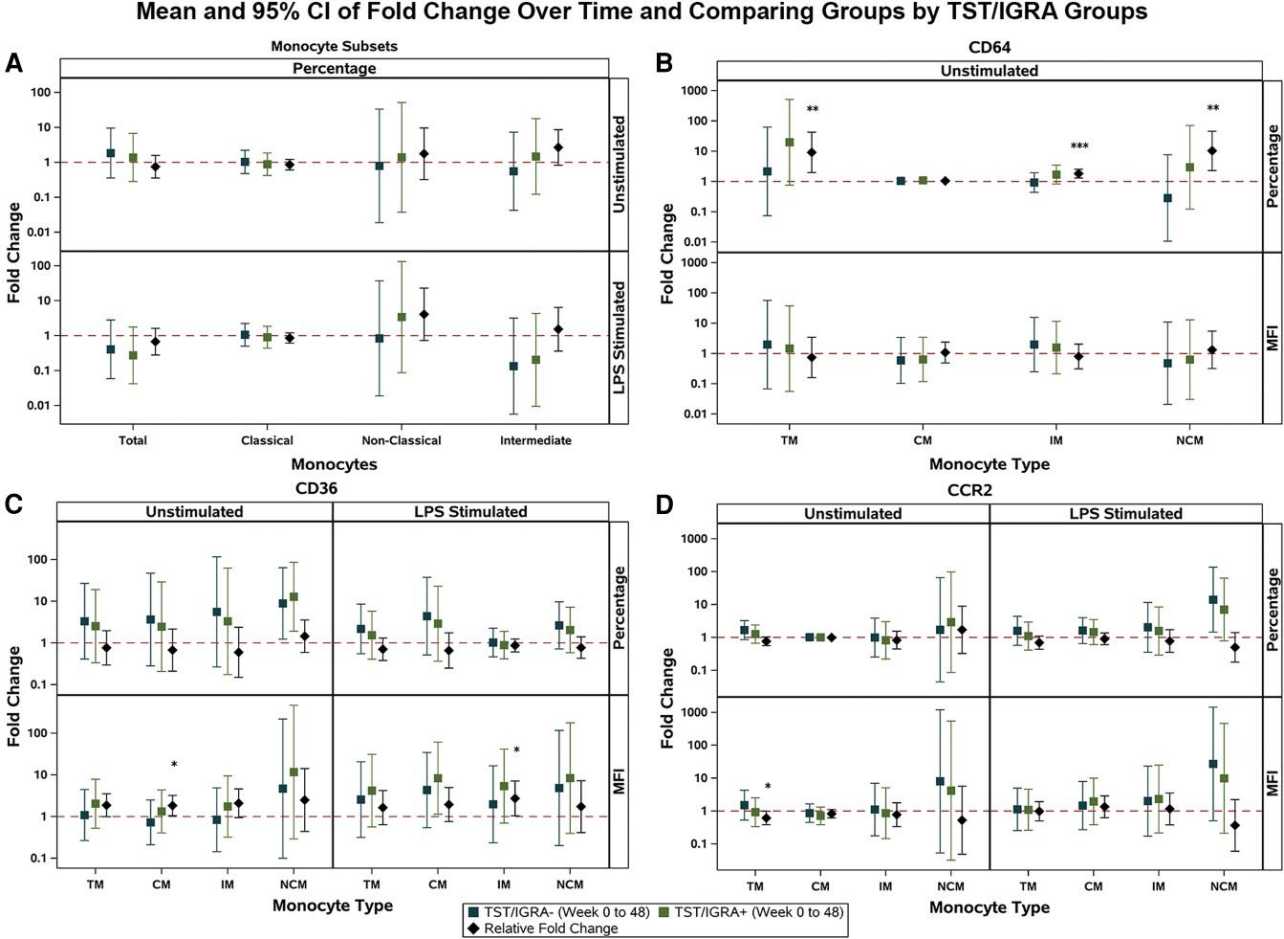
Analyses of the fold changes over time comparing monocyte subsets and CD64, CD36, and CCR2 expression on total monocytes and monocyte subsets between the TST/IGRA-positive and TST/IGRA-negative groups using multivariable linear regression models adjusted for age, sex at birth, country, and CD4+ count. The reference group for the relative fold change is TST/IGRA-negative. Sample size: *n* = 49. Fold changes over time and relative fold changes comparing groups: (*A*) percentage of monocyte subsets; unstimulated and after LPS stimulation; (*B*) percentage and MFI of CD64 expression; unstimulated; (*C*) percentage and MFI of CD36 expression; unstimulated and after LPS stimulation; (*D*) percentage and MFI of CCR2 expression; unstimulated and after LPS stimulation. Abbreviations: MFI, median fluorescence intensity; LPS, lipopolysaccharide; TM, total monocytes; CM, classical monocytes; IM, intermediate monocytes; NCM, nonclassical monocytes.

Paired week 0 (pre-TPT) and week 48 (post-TPT) samples were available from 29 TST/IGRA-positive individuals and 20 TST/IGRA-negative individuals for adjusted analyses of fold changes in monocyte marker expression from pre-TPT to post-TPT. In adjusted analyses, we did not observe significant changes over time in the percentage of total monocytes or monocyte subsets by TST/IGRA group (Figure 2*A*). In the adjusted analyses of monocyte activation markers, we observed higher significant relative fold changes in the percentage of CD64, MFI of CD36, and MFI of CCR2 (Figure 2*B*–*D*) on some monocyte subsets. The differences observed were driven by increases over time in the TST/IGRA-positive group for CD64 (Figure 2*B*) and CD36 (Figure 2*C*) expression, while the TST/IGRA-negative group had increases over time for CCR2 expression on their total monocytes (Figure 2*D*). After in vitro stimulation with LPS, we observed significant relative fold changes in MFI of CD36 in intermediate monocyte expression, driven by increases over time in the TST/IGRA-positive group compared with TST/IGRA-negative (Figure 2*C*). No consistent changes by TST/IGRA status were observed in monocyte IL-6 or TNF-α production.

### Among TST/IGRA-positive Individuals, 1HP Treatment was Associated With an Attenuated Proinflammatory Monocyte Profile Compared With 9H Treatment

We then explored the effects of 1HP and 9H regimens in adjusted analyses restricted to TST/IGRA-positive individuals. Participants assigned to the 1HP arm (*n* = 13) had a significant reduction in the percentage of total monocytes compared with participants in the 9H arm (*n* = 16) over 48 weeks.

**Figure 3.**
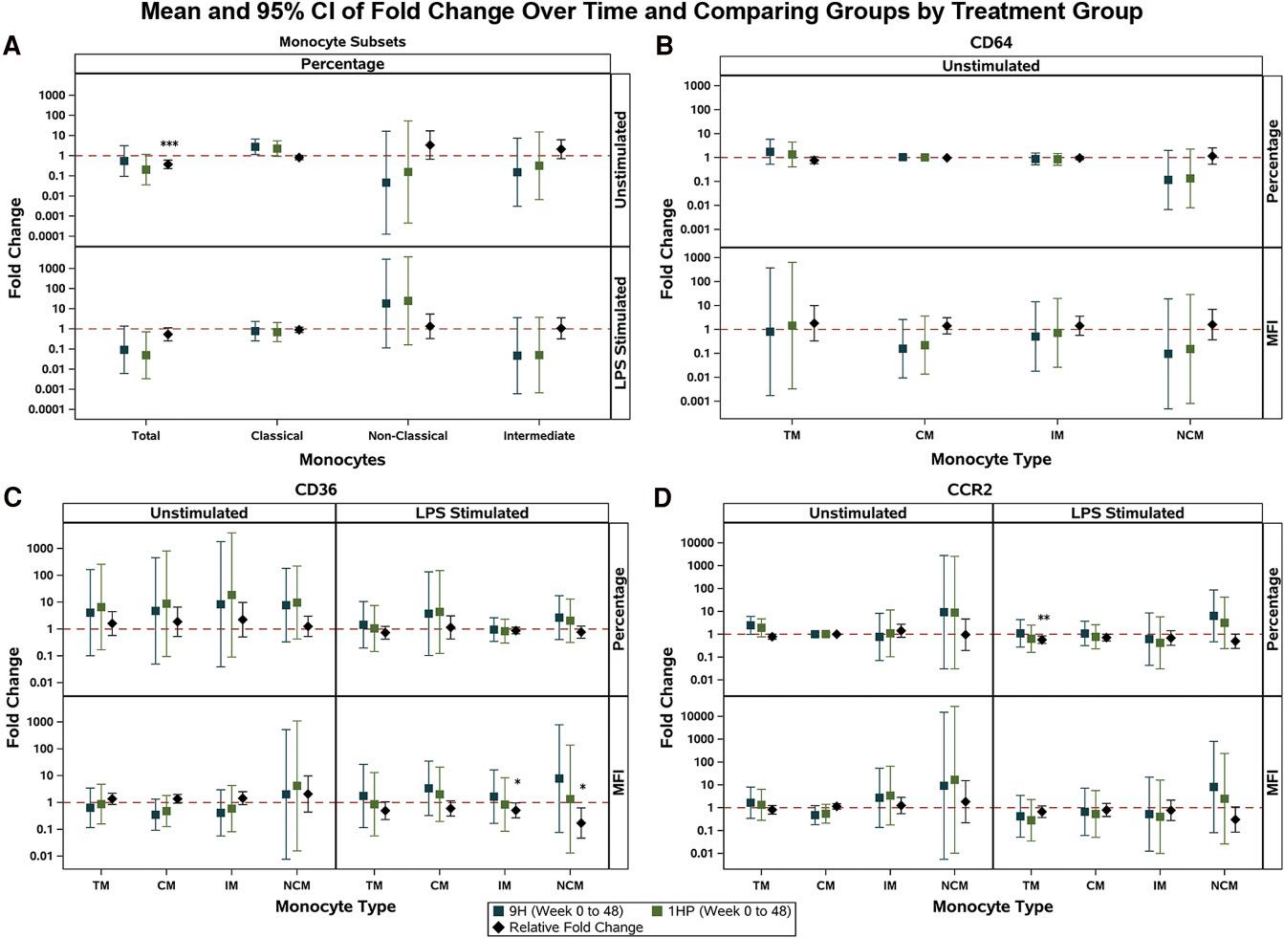
Analyses of the fold changes over time comparing monocyte subsets and CD64, CD36, and CCR2 expression on total monocytes and monocyte subsets between treatment groups in TST/IGRA-positive individuals using multivariable linear regression models adjusted for age, sex at birth, country, and CD4+ count. The reference group for the relative fold change is 9H. Sample size: *n* = 29. Fold changes over time and relative fold changes comparing groups for: (*A*) percentage of monocyte subsets; unstimulated and after LPS stimulation; (*B*) percentage and MFI of CD64 expression; unstimulated; (*C*) percentage and MFI of CD36 expression; unstimulated and after LPS stimulation; (*D*) percentage and MFI of CCR2 expression; unstimulated and after LPS stimulation. Abbreviations: MFI, median fluorescence intensity; LPS, lipopolysaccharide; TM, total monocytes; CM, classical monocytes; IM, intermediate monocytes; NCM, nonclassical monocytes.

**Figure 4.**
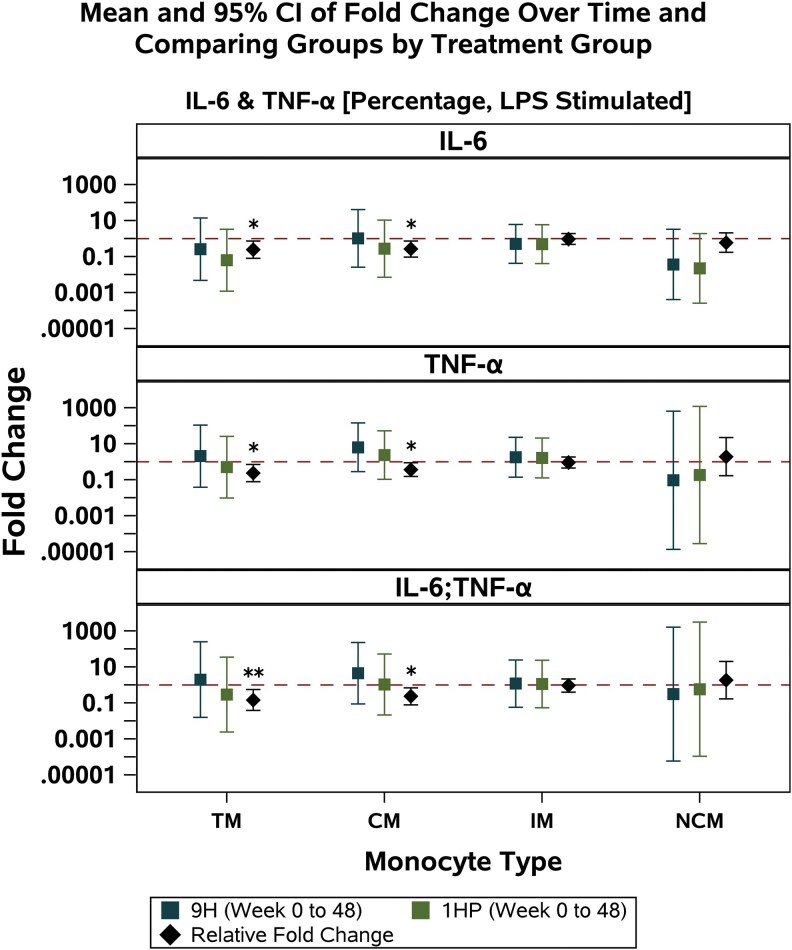
Analyses of the fold changes over time comparing the percentages of positive cells for IL-6, TNF-α, and double-positive IL-6 and TNF-α (assessed by intracellular cytokine staining of total monocytes and monocyte subsets after 6-h LPS stimulation) between treatment groups in TST/IGRA-positive individuals using multivariable linear regression models adjusted for age, sex at birth, country, and CD4+ count. The reference group for the relative fold change is 9H. Sample size: *n* = 29. Abbreviations: IL-6, interleukin-6; TNF-α, tumor necrosis factor-α; LPS, lipopolysaccharide; TM, total monocytes; CM, classical monocytes; IM, intermediate monocytes; NCM, nonclassical monocytes.

Overall, there was a significant decline in the percentage of total monocytes in the 1HP arm compared with 9H (Figure 3*A*). Only after in vitro stimulation with LPS, did we observe difference in monocyte markers and cytokine production between treatment groups. There were significant relative fold changes in the percentage of CCR2+ total monocytes and the density (MFI) of CD36 on some monocyte subsets (Figure 3*C* and 3*D*). These differences between treatment arms were driven by increases in the 9H arm, with minimal changes in the 1HP arm over time (Figure 3*C* and 3*D*). Within total monocytes and the classical monocyte subset, we observed blunted IL-6, TNF-α, and IL-6/TNF-α responses with 1HP compared with 9H (Figure 4).

## DISCUSSION

Compared with PWH with negative TST/IGRA at A5279 study entry, PWH with a positive TST or IGRA exhibited some monocyte alterations indicative of persistent activation pre- and post-TPT. Alterations in the expression of monocyte receptors CD64, CD36, and CCR2 could contribute to the development of inflammation-driven comorbidities. Interestingly, our longitudinal analyses revealed that PWH with a positive TST/IGRA who received the ultra-short 1HP TPT regimen had blunted monocyte proinflammatory cytokine responses (IL-6 and TNF-α), compared with the traditional TPT of 9 months of daily isoniazid. To our knowledge, this is the first study to comprehensively evaluate how TPT influences monocyte activation parameters in PWH, including 1HP.

Our findings are consistent with prior reports of increased monocyte activation in populations with a positive TST or IGRA but without HIV coinfection. A study in Peru showed that monocytes from individuals with a positive IGRA exhibited increased expression of the chemokine receptor CX3CR1 as well as the lipid scavenger receptor CD36 [7]. Our observations suggest distinct monocyte alterations in the setting of HIV, including increased expression of the chemokine receptor CCR2, the activation marker CD64, as well as CD36. We previously reported increased HLA-DR expression on monocytes of IGRA-positive individuals with and without HIV in Uganda [8]; however, differences in sample conditions (fresh PBMCs vs cryopreserved PBMCs), study design, and populations could explain the lack of consistency on HLA-DR findings between the 2 studies. Interestingly, we observed trends toward higher percentage of CD16+ monocytes (eg, CD14^dim^CD16^+^ intermediate and CD14 ^+^ CD16^+^ nonclassical monocytes) in the TST/IGRA-negative group, although these trends were not significant in adjusted analyses. We did not observe significant changes in percentages of CD16+ monocytes over time. Increased percentages of CD16+ monocytes have been associated with enhanced immune activation and cardiovascular disease risk in PWH on ART [14]. Future studies can further investigate the potential effects of TB infection on monocyte subsets distributions.

CCR2 is a chemokine receptor involved in recruitment of monocytes into tissues during infection and other inflammatory conditions [15–17]. Upon CCR2 binding by its ligand CCL2/MCP-1 (monocyte-chemoattractant-1), there is induction of intracellular protein kinases (eg, PKC and MAPK), inositol triphosphate formation, and release of calcium which facilitate cell activation and migration [16]. Modulation of CCR2-mediated monocyte trafficking is under investigation as a potential therapeutic strategy to reduce arterial inflammation in PWH [18, 19]. Our observation of increased CCR2 expression on monocytes from PWH with a positive TST/IGRA may inform future CCR2 immunomodulatory trials, particularly for areas where HIV/*Mtb* coinfection is prevalent. Interestingly, our findings suggest that 1HP TPT leads to blunted monocyte production of IL-6 and TNF-α upon LPS stimulation. Prior studies have demonstrated that IL-6 and TNF-α soluble markers in plasma are robust predictors of morbid outcomes in PWH, including cardiovascular events [20, 21]. Furthermore, in the REPREIVE trial, IL-6 was independently associated with coronary atherosclerosis in PWH [22].

CD64 is a high-affinity Fcγ receptor found in activated monocytes and macrophages. Increased expression of CD64 on monocytes has been documented during active tuberculosis [23], as well as during inflammatory conditions such as metabolic syndrome, rheumatoid arthritis, and systemic lupus erythematosus [24–26]. CD64 ligation on monocytes/macrophages promotes NOD-like receptor protein 3 (NLRP3) inflammasome formation via activation of nuclear factor kappa B (NF-κB) signaling [27], which may further contribute to a proinflammatory state.

We also observed increased expression of CD36 on monocytes from PWH with a positive TST/IGRA, similar to our previous finding among IGRA-positive individuals without HIV [7]. CD36 is a scavenger receptor that allows entry of oxidized lipids into cells [28, 29]. Studies have shown that *Mtb* reprograms monocytes/macrophages to facilitate lipid uptake and induce foamy cell formation for mycobacterial survival, as lipids are a main source of carbon energy for *Mtb* [30, 31]. Such intracellular dysregulation in lipid metabolism induced by mycobacterial infection could favor inflammation and atherogenesis, and may be a potential target for novel therapeutics [32–35].

*M. tuberculosis* replication is increased in HIV coinfection and vice versa [36]. Therefore, it is plausible that *Mtb*-related dysregulation of monocytes may be more prominent in the setting of HIV coinfection and thus contribute to the development of inflammatory comorbidities. Monocyte activation has a key role in the pathogenesis of cardiovascular diseases in PWH. Several studies have shown that systemic inflammation and monocyte activation markers predict future risk of cardiovascular events in PWH [37–39]. We previously showed that having a positive IGRA is associated with increased odds of myocardial infarction among people without HIV in Peru, a tuberculosis-prevalent setting [40]. Furthermore, in a population of people with and without HIV, having a positive IGRA was associated with increased presence of subclinical obstructive coronary artery disease [41]. Future studies are needed to address whether tuberculosis infection influences risk of clinical cardiovascular events in PWH.

Our study had limitations. We recognize that there are several factors besides TST/IGRA status as a marker of *Mtb* exposure/infection that could influence immune activation in PWH. Because uncontrolled HIV infection is a major potential confounder, our analysis focuses on PWH taking ART with a resulting undetectable viral load at time of study entry. Therefore, our findings cannot be generalized to PWH who have not achieved HIV virologic suppression on ART. Although we were unable to adjust for all potential confounders in multivariable models due to sample size, our models accounted for major confounding factors including age, sex at birth, country of origin, and CD4+ T cell count. Regarding the latter, we noticed that positive TST/IGRA results were associated with higher CD4+ T cells counts, which is expected since the sensitivity of TST and IGRA testing is dependent on optimal T cell responses. Although these differences in CD4+ T cell counts by TST/IGRA status could introduce bias, our adjusted analyses demonstrated that CD4+ T cell counts were not associated with differential expression of monocyte activation markers at weeks 0 and 48 in our study population. The time elapsed between blood draws for pre-TPT and post-TPT comparisons is relatively long (48 weeks), particularly for the group that received 1HP. Therefore, we were unable to assess transient changes in monocyte activation markers immediately after completion of each TPT regimen. Finally, we recognize that TST/IGRA testing can have limited sensitivity to identify prior *Mtb* exposure and TB infection, particularly among PWH with low CD4+ T cell counts, and therefore false negative TST/IGRA results are possible. As novel diagnostic assays for TB infection are developed and become available, future studies might be able to minimize risk of potential misclassification.

In conclusion, compared with PWH with negative TST/IGRA, PWH with a positive TST or IGRA exhibited some signals of increased monocyte activation pre- and post-TPT. The 1HP shorter treatment regimen led to a more attenuated proinflammatory monocyte profile compared with treatment with 9H. These novel data may be taken into consideration when implementing TPT strategies in PWH. The observed monocyte alterations in individuals with a positive TST or IGRA may have implications in the pathogenesis of inflammation-driven comorbidities in PWH.

## Supplementary Material

ofaf771_Supplementary_Data

## Supplementary Data

Supplementary materials are available at *Open Forum Infectious Diseases* online. Consisting of data provided by the authors to benefit the reader, the posted materials are not copyedited and are the sole responsibility of the authors, so questions or comments should be addressed to the corresponding author.
