# Supplementary material for: Monocyte Activation in People With HIV and Tuberculosis Coinfection and Effect of Tuberculosis Preventive Therapy: An Analysis of the ACTG A5279/BRIEF TB Trial
Source: Open Forum Infect Dis. 2025 Dec 15;13(1):ofaf771. doi: 10.1093/ofid/ofaf771 (PMC12757864; doi:10.1093/ofid/ofaf771)
Supplement: ofaf771_Supplementary_Data [file ofaf771_supplementary_data.docx]

**SUPPLEMETARY MATERIAL**

**Supplementary Table 1.** Surface mouse anti-human antibodies*

| Marker | Clone | Fluorochrome | Company |
| --- | --- | --- | --- |
| Anti-CD3 | SK7 | PerCP-Cy5.5 | Biolegend |
| Anti-CD14 | HCD14 | APC-Cy7 | Biolegend |
| Anti-CD16 | 3G8 | Brilliant Violet 785 | Biolegend |
| Anti-CD36 | 5-271 | APC | Biolegend |
| Anti-CD56 | HCD56 | Brilliant Violet 421 | Biolegend |
| Anti-CD64 | MD22 | Brilliant Violet 650 | BD |
| Anti-CD80 | 2D10 | PE | Biolegend |
| Anti-CD163 | GHI/61 | Brilliant Violet 711 | Biolegend |
| Anti-CD86 | 2332 (FUN-1) | Brilliant Violet 605 | BD |
| Anti-CX3CR1 | 2A9-1 | Brilliant Violet 510 | Biolegend |
| Anti-CD80 | 2D10 | PE | Biolegend |
| Anti-HLA-DR | LN3 | Alexa Fluor 488 | Biolegend |
| Anti-CCR2 | K036C2 | PE-Cy7 | Biolegend |

***** For stimulation assays, in addition to the above markers we used: **anti-IL6** (clone: MQ2-12A5; fluorochrome: Alexa Fluor 700; Company: eBioscience), and **anti-TNF-α** (clone: Mab11; fluorochrome: Brilliant Violet 650 [in replacement of anti-CD64]; Company: Biolegend).

**Supplementary Table 2.** Analysis of expression of surface markers on **unstimulated** total monocytes and monocyte subsets between TST/IGRA-positive and TST/IGR-negative groups at **week 0**. These unadjusted comparisons between groups used the Wilcoxon rank sum test.

| **Monocytes** | **Marker** | **Percentage: Median (Q1, Q3)** | | | **Median fluorescence intensity (MFI): Median (Q1, Q3)** | | |
| --- | --- | --- | --- | --- | --- | --- | --- |
|  |  | **TST/IGRA-Positive** | **TST/IGRA-Negative** | ***p* value** | **TST/IGRA-Positive** | **TST/IGRA-Negative** | ***p* value** |
| Total | CCR2 | 84.90 (74.50, 89.60) | 79.35 (69.50, 87.60) | 0.18 | 7,690 (5,557, 9,585) | 6,263 (4,753, 8,231) | 0.021 |
| Classical | CCR2 | 98.05 (95.40, 98.80) | 97.80 (97.30, 98.80) | 0.75 | 10,930 (10,175, 12,541) | 9,427 (7,030, 10,615) | 0.0005 |
| Intermediate | CCR2 | 51.25 (43.80, 68.90) | 68.50 (59.20, 76.70) | 0.031 | 3,479 (2,882, 6,579) | 3,882 (2,513, 5,365) | 0.49 |
| Non-classical | CCR2 | 5.77 (2.95, 12.50) | 5.61 (2.62, 27.80) | 0.32 | 120 (83, 227) | 161 (91, 373) | 0.21 |
| Total | CD163 | 48.60 (38.10, 57.10) | 41.60 (28.60, 47.20) | 0.035 | 520 (264, 929) | 685 (474, 1,078) | 0.095 |
| Classical | CD163 | 62.85 (53.60, 68.60) | 70.70 (54.70, 80.00) | 0.044 | 604 (346, 1,111) | 920 (599, 1,129) | 0.12 |
| Intermediate | CD163 | 58.80 (47.20, 74.90) | 68.30 (58.70, 78.30) | 0.054 | 1,401 (923, 1,863) | 1,834 (940, 2,572) | 0.18 |
| Non-classical | CD163 | 25.45 (18.00, 36.60) | 24.15 (14.30, 41.60) | 0.64 | 358 (229, 661) | 196 (41, 367) | 0.013 |
| Total | CD36 | 94.35 (91.90, 95.70) | 92.25 (87.90, 93.90) | 0.033 | 25,109 (16,678, 30,658) | 23,922 (13,698, 29,164) | 0.56 |
| Classical | CD36 | 99.50 (98.20, 99.80) | 99.30 (98.20, 99.80) | 0.46 | 30,877 (20,794, 41,000) | 32,830 (17,001, 41,365) | 0.88 |
| Intermediate | CD36 | 95.80 (91.70, 98.60) | 97.75 (95.90, 99.00) | 0.057 | 19,557 (12,934, 30,102) | 23,854 (13,585, 30,229) | 0.53 |
| Non-classical | CD36 | 57.55 (49.50, 72.50) | 64.65 (50.80, 80.60) | 0.27 | 2,902 (1,395, 4,338) | 2,750 (1,933, 10,020) | 0.30 |
| Total | CD64 | 79.85 (73.00, 86.60) | 55.15 (17.30, 87.60) | 0.017 | 5,144 (4,092, 6,782) | 4,036 (2,830, 5,396) | 0.028 |
| Classical | CD64 | 99.05 (97.50, 99.70) | 98.40 (95.10, 99.40) | 0.13 | 7,464 (6,172, 8,366) | 5,653 (4,428, 6,817) | 0.0092 |
| Intermediate | CD64 | 95.05 (89.70, 97.70) | 94.00 (83.70, 97.90) | 0.62 | 5,400 (4,487, 7,545) | 4,995 (3,268, 6,421) | 0.11 |
| Non-classical | CD64 | 62.90 (41.00, 72.30) | 46.15 (17.30, 55.70) | 0.0018 | 1,128 (972, 1,752) | 970 (595, 1,256) | 0.075 |
| Total | CD80 | 65.55 (55.70, 71.70) | 71.45 (58.60, 75.80) | 0.28 | 338 (265, 384) | 353 (242, 392) | 0.88 |
| Classical | CD80 | 76.15 (64.40, 83.90) | 72.40 (64.20, 84.30) | 0.45 | 325 (270, 392) | 393 (183, 427) | 0.57 |
| Intermediate | CD80 | 75.90 (67.90, 83.30) | 71.10 (49.80, 88.60) | 0.63 | 417 (311, 657) | 376 (207, 529) | 0.12 |
| Non-classical | CD80 | 44.75 (34.00, 52.00) | 63.75 (53.70, 80.00) | < 0.0001 | 215 (175, 245) | 223 (171, 277) | 0.74 |
| Total | CD86 | 87.05 (77.20, 92.70) | 89.75 (71.90, 94.70) | 0.62 | 2,985 (2,715, 3,802) | 3,750 (3,360, 4,014) | 0.015 |
| Classical | CD86 | 98.60 (97.40, 99.30) | 98.90 (95.90, 99.30) | 0.88 | 3,733 (3,169, 4,262) | 4,390 (3,593, 5,424) | 0.0069 |
| Intermediate | CD86 | 98.25 (94.20, 99.20) | 98.60 (96.70, 99.20) | 0.46 | 6,422 (5,356, 7,408) | 6,842 (5,771, 8,409) | 0.37 |
| Non-classical | CD86 | 97.00 (92.60, 98.40) | 97.05 (91.10, 99.00) | 0.65 | 4,450 (4,014, 6,076) | 4,882 (4,091, 5,669) | 0.99 |
| Total | CX3CR1 | 38.80 (30.60, 44.90) | 39.50 (36.80, 51.80) | 0.19 | 783 (676, 902) | 763 (636, 943) | 0.84 |
| Classical | CX3CR1 | 35.75 (26.30, 41.80) | 40.65 (31.80, 53.00) | 0.072 | 667 (553, 770) | 627 (542, 671) | 0.37 |
| Intermediate | CX3CR1 | 89.70 (80.40, 95.40) | 86.50 (73.20, 93.20) | 0.24 | 2,038 (1,733, 2,378) | 1,623 (1,307, 2,177) | 0.010 |
| Non-classical | CX3CR1 | 94.60 (91.30, 97.30) | 94.45 (90.50, 98.20) | 0.86 | 2,265 (2,018, 2,581) | 2,018 (1,727, 2,321) | 0.0067 |
| Total | HLA-DR |  |  |  | 9,900 (8,053, 13,129) | 10,720 (9,678, 13,408) | 0.13 |
| Classical | HLA-DR |  |  |  | 10,976 (7,869, 12,693) | 11,399 (8,048, 13,921) | 0.44 |
| Intermediate | HLA-DR |  |  |  | 27,324 (20,517, 36,680) | 25,553 (20,436, 29,739) | 0.31 |
| Non-classical | HLA-DR |  |  |  | 10,784 (8,890, 15,095) | 11,576 (10,068, 14,107) | 0.55 |

**Supplementary Table 3.** Analysis of expression of surface markers on **unstimulated** total monocytes and monocyte subsets between TST/IGRA-positive and TST/IGR-negative groups at **week 48**. These unadjusted comparisons between groups used the Wilcoxon rank sum test.

| **Monocytes** | **Marker** | **Percentage: Median (Q1, Q3)** | | | **Median fluorescence intensity (MFI): Median (Q1, Q3)** | | |
| --- | --- | --- | --- | --- | --- | --- | --- |
|  |  | **TST/IGRA-Positive** | **TST/IGRA-Negative** | ***p* value** | **TST/IGRA-Positive** | **TST/IGRA-Negative** | ***p* value** |
| Total | CCR2 | 81.30 (71.50, 88.80) | 73.65 (64.20, 83.20) | 0.085 | 7,867 (5,952, 9,205) | 6,654 (4,320, 8,125) | 0.14 |
| Classical | CCR2 | 98.10 (96.20, 98.50) | 98.35 (96.40, 99.40) | 0.16 | 11,395 (9,802, 13,283) | 10,145 (7,675, 11,529) | 0.070 |
| Intermediate | CCR2 | 54.40 (39.00, 69.00) | 65.30 (54.90, 73.50) | 0.053 | 3,711 (2,081, 5,284) | 2,890 (1,971, 4,725) | 0.23 |
| Non-classical | CCR2 | 6.74 (3.74, 10.20) | 13.50 (2.63, 25.70) | 0.41 | 135 (69, 203) | 221 (64, 377) | 0.26 |
| Total | CD163 | 53.60 (42.90, 59.00) | 34.90 (18.40, 44.90) | 0.0011 | 672 (353, 1,138) | 1,061 (860, 1,343) | 0.039 |
| Classical | CD163 | 67.00 (55.10, 76.50) | 79.05 (76.10, 83.90) | < 0.0001 | 880 (590, 1,358) | 1,384 (1,190, 1,555) | 0.0036 |
| Intermediate | CD163 | 66.10 (51.90, 75.00) | 71.75 (63.20, 77.70) | 0.14 | 1,405 (881, 2,292) | 1,846 (1,165, 2,221) | 0.42 |
| Non-classical | CD163 | 27.20 (15.20, 43.70) | 34.90 (18.40, 44.90) | 0.55 | 364 (239, 616) | 203 (0, 468) | 0.0062 |
| Total | CD36 | 93.50 (91.80, 95.40) | 90.25 (83.20, 93.00) | 0.0023 | 23,676 (16,525, 32,589) | 17,247 (13,167, 28,530) | 0.067 |
| Classical | CD36 | 99.60 (99.00, 99.80) | 99.35 (98.20, 99.70) | 0.11 | 31,797 (20,714, 40,207) | 23,936 (16,198, 35,475) | 0.16 |
| Intermediate | CD36 | 98.20 (93.90, 99.30) | 97.00 (93.80, 98.60) | 0.43 | 20,938 (14,209, 30,306) | 19,082 (11,039, 27,851) | 0.67 |
| Non-classical | CD36 | 65.00 (45.80, 86.20) | 67.35 (39.10, 80.50) | 0.76 | 3,060 (1,541, 9,574) | 4,624 (1,054, 7,139) | 0.89 |
| Total | CD64 | 80.10 (74.30, 87.40) | 12.30 (8.98, 38.00) | < 0.0001 | 5,146 (4,302, 6,986) | 3,308 (2,026, 3,977) | < 0.0001 |
| Classical | CD64 | 99.10 (98.60, 99.60) | 97.20 (94.50, 98.50) | < 0.0001 | 7,214 (6,233, 8,214) | 4,524 (3,641, 5,729) | < 0.0001 |
| Intermediate | CD64 | 96.90 (95.30, 98.10) | 72.40 (62.00, 87.40) | < 0.0001 | 5,569 (4,286, 7,124) | 3,399 (2,805, 3,882) | < 0.0001 |
| Non-classical | CD64 | 73.90 (50.50, 82.90) | 12.30 (8.98, 38.00) | < 0.0001 | 1,441 (985, 1,943) | 618 (457, 1,025) | < 0.0001 |
| Total | CD80 | 64.40 (51.20, 76.80) | 59.15 (43.30, 76.90) | 0.33 | 298 (226, 501) | 229 (119, 348) | 0.0093 |
| Classical | CD80 | 72.30 (58.70, 85.30) | 58.55 (38.10, 75.60) | 0.0053 | 310 (242, 381) | 225 (101, 363) | 0.013 |
| Intermediate | CD80 | 74.90 (62.90, 85.70) | 58.70 (39.20, 74.30) | 0.018 | 409 (297, 512) | 208 (126, 433) | 0.0006 |
| Non-classical | CD80 | 38.90 (29.90, 60.00) | 72.40 (68.80, 81.70) | < 0.0001 | 194 (168, 273) | 167 (131, 259) | 0.086 |
| Total | CD86 | 85.60 (77.20, 91.40) | 83.50 (67.40, 92.60) | 0.79 | 3,276 (2,517, 4,079) | 3,536 (2,990, 4,031) | 0.48 |
| Classical | CD86 | 98.60 (97.30, 99.20) | 98.10 (96.20, 99.20) | 0.32 | 3,503 (3,048, 4,199) | 4,463 (3,548, 5,264) | 0.0035 |
| Intermediate | CD86 | 97.90 (96.90, 98.70) | 98.65 (97.60, 99.10) | 0.19 | 6,093 (5,259, 8,073) | 6,268 (5,565, 8,022) | 0.77 |
| Non-classical | CD86 | 97.10 (94.10, 98.30) | 93.55 (89.80, 98.50) | 0.13 | 4,574 (3,398, 5,703) | 4,072 (3,371, 5,393) | 0.38 |
| Total | CX3CR1 | 44.90 (33.50, 51.60) | 47.10 (41.10, 50.40) | 0.23 | 879 (756, 991) | 808 (667, 972) | 0.18 |
| Classical | CX3CR1 | 37.80 (29.90, 47.60) | 47.70 (39.70, 64.20) | 0.019 | 694 (600, 805) | 591 (514, 803) | 0.071 |
| Intermediate | CX3CR1 | 95.00 (90.00, 96.50) | 85.50 (80.50, 92.20) | 0.0046 | 2,321 (2,001, 2,618) | 1,910 (1,420, 2,252) | 0.0047 |
| Non-classical | CX3CR1 | 96.50 (92.20, 98.10) | 98.40 (94.80, 99.30) | 0.016 | 2,510 (2,186, 2,836) | 1,920 (1,637, 2,306) | 0.0006 |
| Total | HLA-DR |  |  |  | 11,333 (8,568, 14,557) | 10,823 (8,979, 14,036) | 0.99 |
| Classical | HLA-DR |  |  |  | 11,068 (8,170, 14,190) | 12,330 (8,904, 15,309) | 0.44 |
| Intermediate | HLA-DR |  |  |  | 27,586 (23,434, 37,979) | 24,745 (20,029, 30,034) | 0.055 |
| Non-classical | HLA-DR |  |  |  | 13,234 (8,480, 17,671) | 12,400 (10,152, 14,126) | 0.35 |

**Supplementary Table 4.** Analysis of expression of surface markers on **LPS-stimulated** total monocytes and monocyte subsets between TST/IGRA-positive and TST/IGR-negative groups at **week 0**. These unadjusted comparisons between groups used the Wilcoxon rank sum test.

| **Monocytes** | **Marker** | **Percentage: Median (Q1, Q3)** | | | **Median fluorescence intensity (MFI): Median (Q1, Q3)** | | |
| --- | --- | --- | --- | --- | --- | --- | --- |
|  |  | **TST/IGRA-Positive** | **TST/IGRA-Negative** | ***p* value** | **TST/IGRA-Positive** | **TST/IGRA-Negative** | ***p* value** |
| Total | CCR2 | 66.35 (53.90, 72.70) | 63.80 (42.70, 70.40) | 0.24 | 2,530 (2,037, 2,947) | 1,743 (1,004, 2,402) | 0.0005 |
| Classical | CCR2 | 83.90 (72.70, 89.50) | 79.40 (59.30, 87.90) | 0.094 | 3,693 (3,068, 4,225) | 2,166 (1,570, 3,382) | < 0.0001 |
| Intermediate | CCR2 | 79.95 (64.70, 90.10) | 65.15 (50.00, 78.50) | 0.013 | 4,236 (2,954, 5,315) | 1,951 (1,186, 2,903) | < 0.0001 |
| Non-classical | CCR2 | 55.85 (42.40, 75.40) | 39.55 (21.10, 48.20) | 0.0001 | 2,440 (1,516, 3,710) | 951 (674, 1,156) | < 0.0001 |
| Total | CD163 | 11.45 (7.31, 14.80) | 8.70 (5.75, 12.60) | 0.23 | 178 (58, 366) | 244 (72, 416) | 0.23 |
| Classical | CD163 | 12.80 (8.78, 16.10) | 10.70 (7.72, 14.70) | 0.37 | 102 (0, 181) | 107 (21, 159) | 0.67 |
| Intermediate | CD163 | 32.35 (22.90, 38.00) | 33.45 (24.50, 49.80) | 0.37 | 1,300 (704, 2,702) | 2,079 (956, 3,451) | 0.085 |
| Non-classical | CD163 | 26.25 (13.10, 34.40) | 28.65 (11.60, 43.70) | 0.28 | 852 (424, 1,275) | 1,257 (221, 2,932) | 0.28 |
| Total | CD36 | 74.80 (64.90, 82.20) | 71.30 (57.10, 79.70) | 0.17 | 7,599 (4,511, 9,197) | 3,937 (2,027, 6,672) | 0.0007 |
| Classical | CD36 | 87.15 (76.90, 93.50) | 83.45 (58.80, 89.80) | 0.099 | 11,045 (7,750, 12,097) | 5,234 (2,229, 9,406) | 0.0022 |
| Intermediate | CD36 | 93.50 (85.90, 97.60) | 91.10 (76.00, 95.60) | 0.16 | 13,715 (10,732, 19,607) | 8,037 (3,860, 12,850) | 0.0023 |
| Non-classical | CD36 | 83.30 (66.80, 92.20) | 78.20 (55.90, 88.30) | 0.17 | 7,666 (4,657, 12,636) | 5,487 (2,562, 6,717) | 0.011 |
| Total | CD80 | 9.39 (5.21, 13.70) | 11.90 (9.02, 20.40) | 0.046 | 91 (76, 103) | 92 (82, 98) | 0.97 |
| Classical | CD80 | 10.20 (6.15, 14.50) | 12.90 (9.10, 17.40) | 0.16 | 92 (74, 101) | 88 (79, 100) | > 0.999 |
| Intermediate | CD80 | 24.55 (16.50, 43.00) | 18.05 (12.60, 22.80) | 0.031 | 95 (73, 123) | 101 (74, 146) | 0.67 |
| Non-classical | CD80 | 26.25 (15.10, 44.30) | 14.85 (11.00, 19.70) | 0.0024 | 88 (76, 128) | 78 (52, 102) | 0.15 |
| Total | CD86 | 68.20 (56.50, 73.70) | 71.05 (57.90, 73.80) | 0.97 | 1,698 (1,439, 1,896) | 1,633 (1,404, 2,099) | 0.98 |
| Classical | CD86 | 84.05 (70.40, 91.40) | 82.55 (55.00, 89.50) | 0.24 | 1,964 (1,705, 2,098) | 1,721 (1,447, 2,239) | 0.23 |
| Intermediate | CD86 | 90.65 (80.10, 97.70) | 88.10 (79.80, 93.40) | 0.19 | 4,112 (2,656, 4,759) | 4,030 (3,018, 5,403) | 0.44 |
| Non-classical | CD86 | 84.45 (73.70, 91.80) | 82.85 (70.70, 86.60) | 0.34 | 3,344 (2,601, 4,130) | 3,622 (2,550, 4,928) | 0.34 |
| Total | CX3CR1 | 48.00 (34.70, 54.80) | 56.55 (47.10, 67.60) | 0.0092 | 724 (570, 801) | 838 (761, 1,053) | 0.0040 |
| Classical | CX3CR1 | 40.20 (28.70, 44.90) | 39.00 (32.60, 50.50) | 0.55 | 690 (603, 788) | 792 (688, 1,083) | 0.014 |
| Intermediate | CX3CR1 | 57.50 (49.70, 68.60) | 69.00 (52.10, 78.90) | 0.12 | 855 (769, 1,158) | 1,289 (796, 1,669) | 0.017 |
| Non-classical | CX3CR1 | 72.30 (61.10, 79.70) | 69.75 (65.10, 79.00) | 0.98 | 1,179 (1,035, 1,512) | 1,391 (1,083, 1,614) | 0.16 |
| Total | HLA-DR |  |  |  | 8,496 (6,381, 10,053) | 8,548 (7,049, 9,917) | 0.76 |
| Classical | HLA-DR |  |  |  | 9,375 (6,506, 11,329) | 8,329 (6,356, 10,798) | 0.56 |
| Intermediate | HLA-DR |  |  |  | 9,507 (6,877, 12,518) | 7,461 (6,148, 10,545) | 0.43 |
| Non-classical | HLA-DR |  |  |  | 10,524 (8,124, 12,239) | 9,020 (6,512, 11,199) | 0.086 |
| Total | IL-6 | 21.30 (14.20, 31.90) | 23.60 (14.60, 30.50) | 0.60 |  |  |  |
| Classical | IL-6 | 41.45 (29.60, 54.60) | 43.45 (16.40, 53.00) | 0.54 |  |  |  |
| Intermediate | IL-6 | 59.90 (41.10, 71.00) | 49.15 (31.40, 71.10) | 0.18 |  |  |  |
| Non-classical | IL-6 | 28.95 (16.40, 39.40) | 22.90 (16.30, 33.40) | 0.57 |  |  |  |
| Total | TNF-α | 27.60 (15.20, 39.00) | 28.90 (15.30, 35.80) | 0.82 |  |  |  |
| Classical | TNF-α | 51.90 (36.90, 63.40) | 48.50 (30.10, 59.10) | 0.24 |  |  |  |
| Intermediate | TNF-α | 72.90 (52.60, 79.70) | 64.85 (44.90, 81.60) | 0.54 |  |  |  |
| Non-classical | TNF-α | 23.20 (7.39, 47.00) | 34.00 (15.00, 52.70) | 0.26 |  |  |  |

**Supplementary Table 5.** Analysis of expression of surface markers on **LPS-stimulated** total monocytes and monocyte subsets between TST/IGRA-positive and TST/IGR-negative groups at **week 48**. These unadjusted comparisons between groups used the Wilcoxon rank sum test.

| **Monocytes** | **Marker** | **Percentage: Median (Q1, Q3)** | | | **Median fluorescence intensity (MFI): Median (Q1, Q3)** | | |
| --- | --- | --- | --- | --- | --- | --- | --- |
|  |  | **TST/IGRA-Positive** | **TST/IGRA-Negative** | ***p* value** | **TST/IGRA-Positive** | **TST/IGRA-Negative** | ***p* value** |
| Total | CCR2 | 64.70 (58.10, 72.90) | 53.10 (40.60, 63.70) | 0.004 | 2,548 (2,218, 3,007) | 1,341 (792, 1,627) | < 0.0001 |
| Classical | CCR2 | 87.75 (77.30, 91.80) | 73.40 (52.20, 80.90) | 0.0003 | 3,745 (3,270, 4,650) | 1,867 (989, 2,202) | < 0.0001 |
| Intermediate | CCR2 | 79.80 (66.50, 89.60) | 69.40 (41.00, 88.10) | 0.13 | 4,127 (3,382, 5,150) | 1,786 (843, 2,405) | < 0.0001 |
| Non-classical | CCR2 | 44.90 (32.30, 58.50) | 33.80 (21.00, 45.10) | 0.023 | 1,578 (1,097, 2,704) | 991 (450, 1,524) | 0.0012 |
| Total | CD163 | 11.80 (10.20, 15.60) | 14.40 (8.86, 21.80) | 0.77 | 233 (152, 404) | 463 (350, 602) | 0.0028 |
| Classical | CD163 | 16.70 (12.80, 21.40) | 16.80 (9.80, 30.30) | 0.90 | 137 (60, 263) | 229 (107, 354) | 0.12 |
| Intermediate | CD163 | 34.90 (28.30, 45.10) | 60.50 (30.60, 76.20) | 0.013 | 1,617 (1,147, 2,706) | 3,157 (2,021, 4,389) | 0.012 |
| Non-classical | CD163 | 30.35 (25.50, 36.20) | 54.50 (36.80, 66.30) | < 0.0001 | 1,069 (744, 1,556) | 2,343 (1,183, 3,300) | 0.0010 |
| Total | CD36 | 74.60 (70.70, 80.20) | 66.40 (52.80, 70.60) | 0.0006 | 8,719 (6,207, 9,727) | 2,810 (1,670, 3,263) | < 0.0001 |
| Classical | CD36 | 90.75 (81.60, 94.10) | 79.30 (64.30, 86.90) | 0.0007 | 11,862 (9,547, 14,080) | 3,511 (2,222, 4,997) | < 0.0001 |
| Intermediate | CD36 | 95.30 (89.10, 97.80) | 94.00 (74.50, 95.80) | 0.11 | 16,480 (11,806, 20,861) | 5,937 (2,673, 7,180) | < 0.0001 |
| Non-classical | CD36 | 79.30 (67.00, 85.20) | 74.90 (65.00, 81.60) | 0.44 | 7,843 (4,965, 9,737) | 3,834 (1,751, 5,985) | 0.0009 |
| Total | CD80 | 8.98 (7.15, 12.90) | 15.80 (12.10, 17.90) | 0.0004 | 88 (80, 99) | 82 (80, 91) | 0.22 |
| Classical | CD80 | 9.01 (7.09, 13.90) | 12.70 (8.19, 17.90) | 0.21 | 85 (77, 98) | 87 (72, 96) | 0.59 |
| Intermediate | CD80 | 25.35 (19.30, 35.20) | 10.70 (8.91, 21.50) | 0.0013 | 85 (64, 106) | 111 (89, 154) | 0.0080 |
| Non-classical | CD80 | 25.60 (18.20, 36.10) | 13.50 (7.25, 19.70) | 0.0007 | 88 (64, 111) | 79 (62, 96) | 0.45 |
| Total | CD86 | 67.30 (64.10, 74.90) | 67.20 (60.70, 71.90) | 0.29 | 1,869 (1,638, 2,142) | 1,693 (1,492, 2,009) | 0.26 |
| Classical | CD86 | 87.20 (77.90, 92.40) | 83.80 (67.90, 86.90) | 0.022 | 2,168 (1,835, 2,467) | 1,665 (1,466, 2,160) | 0.017 |
| Intermediate | CD86 | 94.05 (87.70, 96.50) | 92.20 (83.90, 94.20) | 0.16 | 4,103 (3,189, 4,842) | 4,906 (3,945, 7,033) | 0.028 |
| Non-classical | CD86 | 84.70 (73.10, 88.00) | 82.60 (73.80, 90.40) | 0.86 | 3,446 (2,971, 4,038) | 4,576 (3,525, 5,505) | 0.025 |
| Total | CX3CR1 | 50.40 (38.10, 58.40) | 63.40 (59.50, 69.80) | 0.0006 | 732 (637, 868) | 970 (858, 1,130) | 0.0007 |
| Classical | CX3CR1 | 40.60 (26.10, 48.60) | 46.30 (37.60, 59.40) | 0.092 | 710 (568, 800) | 973 (838, 1,164) | < 0.0001 |
| Intermediate | CX3CR1 | 54.00 (46.10, 66.90) | 75.00 (68.80, 84.50) | < 0.0001 | 886 (762, 1,043) | 1,419 (1,313, 1,841) | < 0.0001 |
| Non-classical | CX3CR1 | 68.35 (62.80, 77.90) | 76.60 (67.70, 81.30) | 0.082 | 1,284 (1,064, 1,490) | 1,578 (1,314, 1,754) | 0.013 |
| Total | HLA-DR |  |  |  | 9,086 (6,988, 11,245) | 8,585 (7,761, 9,973) | 0.57 |
| Classical | HLA-DR |  |  |  | 9,126 (7,020, 10,636) | 8,351 (7,393, 10,592) | 0.89 |
| Intermediate | HLA-DR |  |  |  | 10,564 (7,538, 12,978) | 8,492 (7,377, 11,817) | 0.30 |
| Non-classical | HLA-DR |  |  |  | 11,327 (8,705, 13,088) | 8,785 (6,870, 11,024) | 0.050 |
| Total | IL-6 | 25.60 (20.30, 34.90) | 28.80 (21.10, 35.00) | 0.74 |  |  |  |
| Classical | IL-6 | 49.60 (44.00, 63.00) | 47.90 (29.20, 54.80) | 0.098 |  |  |  |
| Intermediate | IL-6 | 61.45 (51.00, 76.50) | 71.00 (48.10, 78.10) | 0.92 |  |  |  |
| Non-classical | IL-6 | 28.35 (18.40, 44.20) | 35.60 (23.00, 52.70) | 0.19 |  |  |  |
| Total | TNF-α | 35.45 (24.30, 40.30) | 30.70 (19.50, 37.80) | 0.11 |  |  |  |
| Classical | TNF-α | 59.75 (47.70, 70.50) | 51.20 (30.20, 57.90) | 0.0083 |  |  |  |
| Intermediate | TNF-α | 78.05 (62.40, 84.00) | 64.10 (51.70, 83.00) | 0.083 |  |  |  |
| Non-classical | TNF-α | 31.50 (24.40, 43.60) | 34.60 (13.70, 50.60) | 0.86 |  |  |  |

**Supplemental Figure 1.** Gating strategy for detecting total monocytes, classical monocytes, intermediate monocytes, and non-classical monocytes. Detection of the inflammatory markers, and receptors in subsets of monocytes.

**
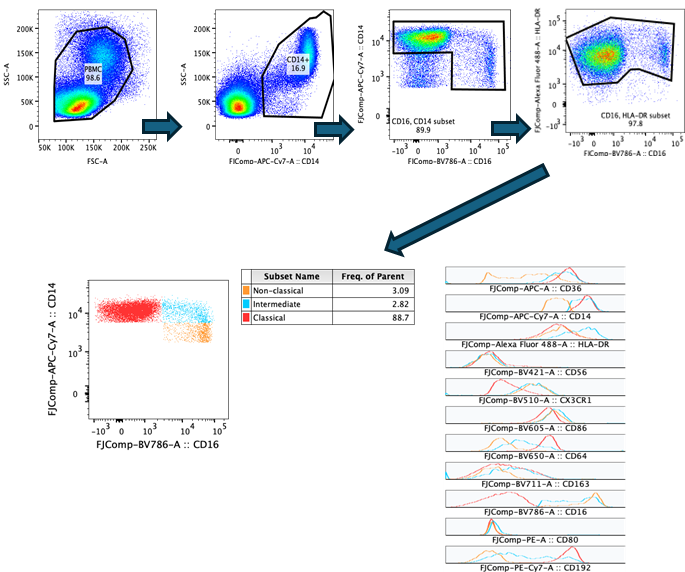
**
